# Similar Strength and Power Adaptations between Two Different Velocity-Based Training Regimens in Collegiate Female Volleyball Players

**DOI:** 10.3390/sports6040163

**Published:** 2018-12-04

**Authors:** Jacob T. Rauch, Irineu Loturco, Nicholas Cheesman, Justin Thiel, Michael Alvarez, Nicholas Miller, Nathan Carpenter, Christopher Barakat, Gloria Velasquez, Alexandria Stanjones, Daniel Aube, Jody C. Andersen, Eduardo O. De Souza

**Affiliations:** 1 Human Performance Laboratory, Health Sciences and Human Performance Department, University of Tampa Florida, Tampa, FL 33606, USA; nicholas.cheesman@spartans.ut.edu (N.C.); JTHIEL@ut.edu (J.T.); michael.alvarez@spartans.ut.edu(M.A.); nicholas.miller@spartans.ut.edu (N.M.); nathan.carpenter@spartans.ut.edu (N.C.); cbarakat@ut.edu (C.B.); gloriavelazquez@yahoo.com (G.V.); alexandria.r.stanjones@gmail.com (A.S.); daniel.aube@spartans.ut.edu (D.A.); jcandersen@ut.edu (J.C.A.); edesouza@ut.edu (E.O.D.S.); 2 Nucleus of High Performance in Sport, Sāo Paulo 03187-010, Brazil; irineu.loturco@terra.com.br

**Keywords:** female athletes, power training, velocity-based training, optimum training load, body composition

## Abstract

This study investigated the effects of two different velocity-based training (VBT) regimens on muscular adaptations. Fifteen female college volleyball players were randomly assigned into either progressive velocity-based training (PVBT) or optimum training load (OTL). Both groups trained three times a week for seven weeks. PVBT performed a 4-week strength block (e.g., 0.55–0.70 m·s^−1^) followed by a 3-week power block (e.g., 0.85–1.0 m·s^−1^), whereas OTL performed training at ~0.85–0.9 m·s^−1^. 1RM and peak power output (PP) assessments on the back squat (BS), bench press (BP) and deadlift (DL) exercises were assessed pre and post training. There was a main time effect (*p* ≤ 0.05) for BS and BP 1RM, (PVBT: 19.6%, ES: 1.72; OTL: 18.3%, ES: 1.57) and (PVBT: 8.5%, ES: 0.58; OTL: 10.2%, ES: 0.72), respectively. OTL increased DL 1RM to a greater extent than PVBT (*p* ≤ 0.05), (OTL: 22.9%, ES: 1.49; PVBT: 10.9%, ES: 0.88). Lastly, there was a main time effect (*p* ≤ 0.05) for BS, BP and DL PP, (PVBT: 18.3%, ES: 0.86; OTL: 19.8%, ES: 0.79); (PVBT: 14.5%, ES: 0.81; OTL: 27.9%, ES: 1.68); (PVBT: 15.7%, ES: 1.32; OTL: 20.1%, ES: 1.77) respectively. Our data suggest that both VBT regimens are effective for improving muscular performance in college volleyball players during the offseason period.

## 1. Introduction

Volleyball is characterized by short bouts of explosive activity including jumps, dives, sprints, spikes, and multi-directional court movements [1]. In addition to technical and tactical skills, it is suggested that muscular strength and power are the most important attributes underlying successful performance [2]. Moreover, lower body strength and power levels have previously been associated with jump performance, a critical performance indicator in volleyball [3]. Therefore, strength and conditioning professionals are constantly looking for effective training regimens to improve muscular strength and power output amongst volleyball athletes.

Traditionally, Strength Training (ST) and Power Training (PT) regimens have been prescribed based on a percentage of an athlete’s one-repetition maximum (1RM). However, recently there have been concerns with percentage-based training, as it is time consuming, requires multiple 1RM assessments, and may increase risk of injury if performed improperly. Additionally, the prescribed intensity may not accurately reflect an athlete’s ability due to daily fluctuations in 1RM performance, which may overload or underload the athlete accordingly [4].

In this regard, velocity-based training (VBT) has been presented as an alternative method to prescribe training intensity, as research has demonstrated an inverse relationship between load and velocity (i.e., as load magnitude increases, velocity of movement decreases) [4,5,6]. Moreover, researchers have identified that each percentage of an athlete’s 1RM has its own velocity (I.E 60% 1RM on the bench press exercise = 0.8 m·s^−1^) [4], further enabling practitioners to target a specific stimulus for a given session. For example, practitioners could prescribe 0.48–0.19 m·s^−1^ (80–100% 1RM) for a strength stimulus or 1.33–0.80 m·s^−1^ (30–60%) for a power stimulus. Another major advantage of VBT is the constant feedback provided to practitioners regarding velocity and power output [7].

While researchers agree on the benefits of using VBT, there is still an ongoing debate as to the most effective VBT progression for simultaneous strength and power development. Two commonly-prescribed models are: progressive overload-VBT (PVBT), and optimum training load (OTL). PVBT starts with high force and lower velocity (i.e., absolute strength <0.55 m·s^−1^), and then gradually progresses into low force, higher velocity (i.e., strength speed 0.75–1.0 m·s^−1^) over the course of a macrocycle [5]. As PVBT increases the rate of force development (i.e., the ability to rapidly develop muscular force) [8] over time, it has been suggested this will allow athletes to continually increase their explosive abilities [5]. Additionally, it has been suggested that the adaptations made in the initial strength phase will enhance the adaptations during the subsequent power phase [9]. In contrast, OTL does not progress the training intensity; rather, it suggests that athletes consistently train with individualized loads capable of maximizing muscle power output (e.g., 0.8–1.0 m·s^−1^) and adjusting the training load as athletes increase or decrease the movement velocity within the “optimum power zones” [10]. Interestingly, despite training with a lower relative intensity (~40–60% 1RM), OTL has been shown to induce improvements at both ends of the force-velocity curve [11].

Although both training methods have been shown to enhance muscular strength and power development, there is a paucity of data comparing different VBT progressions. Therefore, the purpose of our investigation was to assess the effects of two different VBT progressions (PVBT vs. OTL) on muscle strength and power adaptations in female collegiate volleyball players during an off-season mesocycle.

## 2. Materials and Methods

This was a parallel-group, repeated measures design, which examined the effects of two different training methods (PVBT and OTL) on strength and power adaptations in female collegiate volleyball players. Additionally, as body composition can affect performance, lean body mass and fat mass were evaluated via DEXA. All subjects completed six familiarization sessions over the course of two weeks prior to baseline testing. Each subject had two sessions with back squat (BS), bench press (BP), and deadlift (DL) exercises. Familiarization sessions consisted of a 15-min dynamic warm up led by the head strength and conditioning coach, followed by two warm up sets at 50% of their estimated 1RM and five working sets at 65–80% of their estimated 1RM on the respective exercise for that given day. During the working sets, subjects were introduced to the linear position transducer feedback (Tendo Sports Machines, Trencin, Slovak Republic), as well as the proper depth and form that were to be replicated throughout testing. For the BS exercise, subjects were instructed to squat to a parallel hip stance where a medicine ball and weight plates were then placed to standardize depth (i.e., ~100° knee flexion). For the BP exercise, subjects were required to maintain five points of contact (head, shoulder blades, lower back, left foot, and right foot) while lowering the bar, with control touching the sternum and fully extending the arms for a repetition to be considered successful. For the DL exercise, subjects were required to lift the barbell off the ground with control to a fully erect position for a repetition to be considered successful. After familiarization procedures, subjects underwent baseline testing on body composition, jump height, agility (*t-test*), 1RM and submaximal peak power assessments on the BS, BP, and DL exercises over the course of three testing sessions throughout the week. The inter-day variance during familiarization sessions was <5% for all the performance assessments. Subjects were then matched and randomly assigned to their respective groups (i.e., PVBT or OTL) based on 1RM performance on the BS exercise. Weight training took place on Monday, Wednesday, and Friday, and sport-specific team practices were held on Tuesday and Thursday. Both groups trained 3 times a week for 7 weeks and were re-tested during week 8. Subjects received post-workout Dymatize Elite whey protein powder (25 g) after each training session. Additionally, each subject met with a blinded registered dietitian, and received personal counseling to ensure optimal energy intake. Lastly, rate of perceived exertion (RPE) was collected in isolation at the end of each training session. Testing of all dependent variables occurred at weeks 0 and 8. Subjects were required to attend at least 90% of all training sessions to be considered for further analysis.

### 2.1. Subjects

Fifteen female collegiate volleyball players were subjects for this study (age: 19.3 ± 1.4 years, height: 176.5 ± 6.3 cm, lean body mass [LBM]: 50.7 ± 5.5 kg, BS: 1RM 87.5 ± 10.5 kg). Inclusion criteria consisted of being on the volleyball team for the following fall season. Subjects were excluded from participation if they were currently taking any medication or anti-inflammatory drugs. In addition, this study was conducted according to the ethical standards of the Deceleration of Helsinki. All subjects read and signed an informed consent approved by the institutional review board.

### 2.2. Body Composition Assessment

A Lunar Prodigy dual-energy X-ray absorptiometry (DEXA) apparatus (Hologic, Bedford, MA, USA) was used to measure body composition. Subjects were required to fast for 10 h before the examination and refrain from exercising for 48 h before the assessment. Total LBM and fat mass (FM) were determined with the subject lying in a supine position with their knees extended and instructed not to move for the entire duration of the scan. The same researcher, who was blinded for the experimental groups, performed all the DEXA scans. The coefficient of variation (CV) was determined before the study using five different subjects with similar characteristics to the current participants. These DEXA scans were performed on three different days interspersed by 48 h at the same time of the day. The CV for lean body mass was ~1.2%.

### 2.3. Squat Jump and Counter Movement Jump Height

For the counter-movement jump (CMJ) and squat jump (SJ), subjects were instructed to place their hands on their hips and keep them in contact with their hips for the duration of the jump and landing. For the CMJ, subjects were instructed to perform a counter-movement and quickly reverse it to generate vertical momentum [12]. Subjects were instructed to jump as high as possible, and to land with both feet at the same time. For the SJ, subjects were instructed to execute a squat position until the knee joint was at 90° and hold the position for three seconds, as counted out loud by the testers. After three seconds, the testers instructed the subjects to jump as high as possible. Subjects were instructed not to perform any counter-movement from the squat position whatsoever, and if a subject performed a counter-movement (as evidenced by force plate data), the jump was disqualified, and the subject was instructed to perform the jump again after a rest of 30 s. Subjects were instructed to jump as high as possible and to land with both feet at the same time. Each participant completed three jumps per condition, with 30 s of rest between each jump and 2-min of rest between each condition. Jump height was calculated from the force plate using manufacturers software (AccuPower 2.0, Advanced Mechanical Technology Incorporated, Watertown, MA, USA). The highest jump from each trial was used for further analysis. All the jumps were performed on a portable force plate (AMTI^®^, AccuPower, Watertown, MA, USA).

### 2.4. Agility (t-Test)

Agility performance was assessed through the *t*-test, adapted from Haff & Triplett [13]. Briefly, subjects were instructed to start at the base of the *“t”.* Next, following an auditory signal, the subjects would sprint straight 10 yards to the first cone, touch the base of the cone with their right hand, shuffle 5 yards to the left, and touch the base of the cone with their left hand. Subsequently, the subjects would then shuffle 10 yards to the right and touch the base of the cone with their right hand, shuffle 5 yards back to center, followed by a 10-yard backward sprint to the starting point. The examiner would stop the timer once the athlete passed the starting point. The same researcher conducted all assessments. Each athlete was given a warm-up trial followed by two maximal attempts with 5 min rest in between attempts. The best score from the two attempts was used for further analysis.

### 2.5. Maximum Dynamic Strength

Maximum strength was assessed on the BS, BP, and DL exercises following the recommendations of Haff & Triplett [13]. During the first set, participants performed 10 repetitions with 50% of their predicted 1RM. In the second set, they performed five repetitions with 75% of their predicted 1RM. After the second warm-up set, participants rested for three minutes. Next, each participant had up to five attempts to achieve their 1RM load. The 1RM load was considered the maximum weight an athlete could lift with proper form before reaching failure. A rest period of five minutes was allotted between 1RM attempts. Strong verbal encouragement was given throughout the 1RM test.

### 2.6. Muscle Power Output Assessments

Muscle power output assessments were adapted from Loturco et al. [14] Briefly, the subjects were instructed to perform three repetitions with maximum velocity on each set, starting at 40% of their body mass. A load corresponding to 10% of body mass was gradually added in each set until a decrease in peak power was observed in the feedback display. Peak power output on each set was used for further analysis. These assessments were performed for the BS, BP, and DL exercises. For the BS assessment, subjects were instructed to squat to a parallel hip stance where a medicine ball and weight plates were then placed to standardize depth (i.e., ~100° knee flexion). Once depth was achieved, subjects were instructed to perform the concentric phase as fast and explosively as possible while maintaining the bar in contact with their back and feet on the floor. For the BP exercise, subjects were instructed to lower the bar with control up to touching the sternum, and then perform the concentric phase as fast and explosively as possible while maintaining grip of the bar. For the DL exercise, subjects were instructed to grasp the bar in the starting position and then perform the concentric phase as fast and explosively as possible to a fully erect posture, and then drop the bar. Each subject was allowed 3 min of rest between trials. To determine average velocity, a linear position transducer (LPT) Tendo Power Output Unit (Tendo Sports Machines, Trencin, Slovak Republic) was attached to the barbell. LPTs estimate power output by measuring barbell displacement and time. First, velocity is calculated via V = (Δd × Δt^−1^) where d is the displacement in millimeters and t is time in seconds. Next, acceleration is calculated via A = (Δv ×Δt^−1^). Thereafter, force is calculated via F= (m × g) + (m × a), whereby m = the mass of the load on the bar and mass of the barbell and g is the acceleration due to gravity. Lastly, power output in Watts is determined via P = (F × V) [15]. The average speed at which maximum power output was achieved was then used to prescribe the training speeds for the OTL group throughout training. The Tendo Unit LPT (Tendo Sports Machines, Trencin, Slovak Republic) has previously been demonstrated to be both valid and reliable for determining movement velocity and power [16].

### 2.7. Nutritional Assessments

Subjects met with a licensed registered dietitian, who was blinded for the training interventions, once per week throughout the study. Total Kcal, relative fat, carbohydrate, and protein intake (i.e., g/kg^−1^) was quantified at weeks 0 and 7. All dietary information was analyzed for average energy and macronutrient content utilizing (MyfitnessPal—http://www.myfitnesspal.com). In addition, each participant was provided with one serving (25 g) of protein powder immediately after each exercise session (Elite Whey Protein; Dymatize Nutrition, Dallas, TX, USA).

### 2.8. Rate of Perceived Exertion

Rate of perceived exertion (RPE) assessments were performed 5 min after each training session. Subjects were required to point to a number on a 1–10 scale that best indicated their perceived level of effort for that given training session. All assessments were performed in isolation from other subjects to ensure accuracy. RPE of the three weekly sessions were averaged for further analysis [17].

### 2.9. Strength Training Program

Subjects underwent a 7-week (3 sessions per week) experimental period. The sets and velocity progressions for PVBT are displayed in Table 1 and Table 2. Briefly, PVBT performed a 4-week strength block (e.g., 0.55–0.70 m·s^−1^) followed by a 3-week power block (e.g., 0.85–1.0 m·s^−1^). OTL trained at 0.85 m·s^−1^, 0.85 m·s^−1^and 0.9 m·s^−1^on the BS, BP and DL respectively for weeks 1–7. With regard to the OTL training prescription, previous research has shown these speeds to correlate with 67.5% of 1RM on the BS and 55% 1RM on the BP [18]. The load each subject could move in the target speed zone was adjusted for each set on a daily basis. Each workout started with one criterion lift (Monday—BS, Wednesday—BP, Friday—DL) followed by several accessory exercises performed in a circuit fashion (Table 3). A 2-min rest interval was provided at the completion of each circuit. Total volume load per training session, average speed (m·s^−1^), and peak power output (Watts) were recorded for the best repetition in each set throughout the entire experimental period.

### 2.10. Statistical Analysis

After normality (i.e., Shapiro-Wilk) and variance assurance (i.e., Levene), a 2-sample t-test was used to detect differences between groups before training. Total volume load between groups was also compared using a 2-sample t-test. A mixed model approach was used for the remainder of the dependent variables assuming groups (e.g., PVBT and OTL) and time (e.g., pre- and post-testing) as fixed factors and subjects as a random factor. (SAS 9.4; SAS Institute, Inc., Cary, NC, USA). Whenever a significant F value was obtained, a post hoc test with a Tukey’s adjustment was performed for multiple comparison purposes. In addition, 95% confidence intervals for the within-group and between-groups comparisons (CI_diff_) were calculated. Within-group effect sizes (ES) were calculated via: [(M1 − M2)/SD_pooled_)]. The significance level was previously set at *p* ≤ 0.05. Results are expressed as mean ± SD.

## 3. Results

Baseline values were not significantly different between groups for any of our dependent variables (*p* > 0.05). In addition, there were no significant differences in total volume load between PVBT and OTL (82,548.1 ± 15,447.7 kg and 82,808.9 ± 7753.6 kg, *p* = 0.96, respectively).

### 3.1. Body Composition

For LBM, there was a main time effect (*p* ≤ 0.0001) in which both groups increased LBM. (PVBT: 5.3%, CI_diff_: 1.4 to 3.8 kg, ES: 0.54; OTL: 5.4%, CI_diff_: 1.5 to 4.3 kg, ES: 0.26). For FM there was a main time effect (*p* ≤ 0.0001) in which both groups decreased FM. (PVBT: −10.1%, CI_diff_: −3.1 to −0.6 kg, ES: −0.23, OTL: −8.5%, CI_diff_: −3.7 to −0.9 kg, ES: −0.46).

### 3.2. Vertical Jump Assessments and Agility

No significant changes were observed across time points or groups for CMJ and SJ (*p* ≥ 0.05). For agility, there was a main time effect (*p* ≤ 0.043) in which both groups decreased trial times (PVBT: −1.8%, CI_diff_: −0.5 to 0.1 s, ES: −0.40; OTL: −2.1%, CI_diff_: −0.6 to 0.1 s, ES: −0.88).

### 3.3. Maximum Dynamic Strength

**Figure 1:**
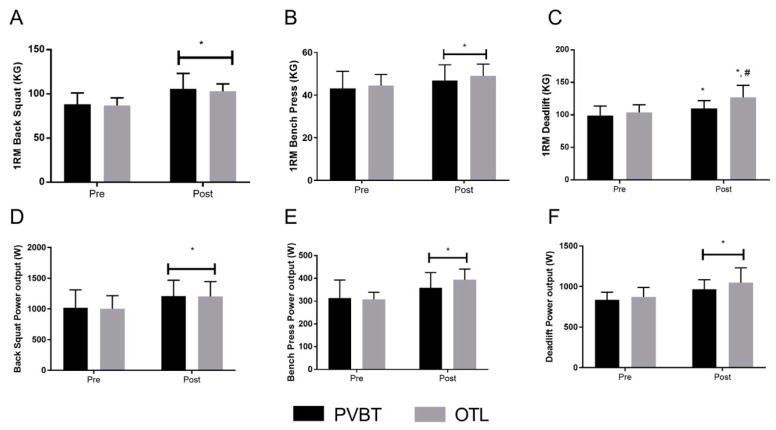
One repetition maximum (Kg) and peak power (Watts) results. (**A**) 1RM back squat; (**B**) 1RM bench press; (**C**) 1RM deadlift; (**D**) back squat peak power; (**E**) bench press peak power; (**F**) deadlift peak power. #—Indicates *p* ≤ 0.05 group by time interaction *—Indicates *p* ≤ 0.05 main time effect; W—Watts; KG—kilograms.

For the BS 1RM, there was a main time effect (*p* ≤ 0.0001) in which both groups increased 1RM values at post testing (PVBT: 19.6%, CI_diff_: 11.0 to 23.6 kg, ES: 1.72; OTL: 18.3%, CI_diff_: 9.2 to 22.6 kg, ES: 1.57), (Figure 1A). For BP 1RM, there was a main time effect (*p* ≤ 0.002) in which both groups increased 1RM values at post testing (PVBT: 8.5%, CI_diff_: −0.04 to 7.3 kg, ES: 0.58; OTL: 10.2%, CI_diff_: 0.5 to 8.5 kg, ES: 0.72), (Figure 1B). For the DL 1RM, there was a group by time interaction (*p* ≤ 0.002) in which OTL increased 1RM to a greater extent than PVBT (PVBT: 10.9%, CI_diff_: 5.4 to 16.1kg, ES: 0.88; OTL: 22.9%, CI_diff_: 16.9 to 29.3 kg, ES: 1.49), (Figure 1C).

### 3.4. Muscle Power Output

For BS peak power output, there was a main time effect (*p* ≤ 0.002) in which both groups increased peak power output (PVBT: 18.3%, CI_diff_: 47.3 to 327.9 Watts, ES: 0.86; OTL: 19.8%, CI_diff_: 88.3 to 310.1 Watts, ES 0.79), (Figure 1D). For BP power output, there was a main time effect (*p* ≤ 0.0001) in which both groups increased peak power output (PVBT: 14.5%, CI_diff_: 6.7 to 84.8 Watts, ES: 0.81; OTL: 27.9%, CI_diff_: 41.0 to 131.2 Watts, ES: 1.68), (Figure 1E). For DL power output, there was a main time effect (*p* ≤ 0.0003) in which both groups increased peak power output (PVBT: 15.7%, CI_diff_: 23.9 to 238.8 Watts, ES: 1.32; OTL: 20.1%, CI_diff_: 61.1 to 290.8 Watts, ES: 1.77), (Figure 1F).

### 3.5. Energy Intake and Macronutrient Distribution

There were no significant differences (*p* ≥ 0.34) for energy intake from pre to post-test (PBVT: 3.3%, CI_diff_: −56.4 to 207.9 Kcal, ES: 0.44; OTL: −5.9%, CI_diff_: −274.3 to 6.3 Kcal, ES: 0.78). For fat intake, there was a significant group effect (*p* ≤ 0.01) in which PVBT consumed more fat than OTL (PVBT vs. OTL: 25.9%, CI_diff_: 0.05 to 0.3 g/kg^−1^, ES: 1.64). For carbohydrates, there was a significant group effect (*p* ≤ 0.01) in which PVBT consumed more carbohydrates than OTL (PVBT vs. OTL: 24.0%, CI_diff_: 0.1 to 1.3 g/kg^−1^, ES: 1.47). For protein, while not statistically significant (*p* ≤ 0.08), PVBT consumed more protein than OTL (PVBT vs. OTL: 15.1%, CI_diff_: 0.03 to 0.5 g/kg^−1^, ES: 1.01).

### 3.6. Rate of Perceived Exertion

There was a significant main time effect (*p* ≤ 0.0001) in which both groups increased RPE from week 1 post-test (PBVT: 37.4%, CI_diff:_ 0.9 to 3.0 AU, ES: 2.01; OTL: 18.4%, CIdiff: 0.06 to 2.3 AU, ES: 1.66).

## 4. Discussion

The purpose of this investigation was to compare the effects of two different VBT regimens (PVBT vs. OTL) on muscle strength and power adaptations in college volleyball athletes. Our findings suggest that both groups demonstrated significant decreases in fat mass and increases in lean body mass (−2.1 kg and 2.7 kg, respectively). Additionally, both groups demonstrated similar strength gains on the BS and BP exercise; however, OTL demonstrated greater strength gains on the DL exercise compared to PVBT. Lastly, both groups demonstrated similar improvements in peak power on the BS, BP, and DL exercises. Therefore, our data suggest that PVBT and OTL are effective methods for improving body composition, strength, and power parameters in college volleyball players during an off-season training period.

Regarding body composition, both groups demonstrated ~2.7 kg lean mass augmentation. While these gains (i.e., ~5.0%) may seem large for an athletic population, it is important to note that there is limited data available analyzing the effects of different ST regimens on LBM adaptations in female athletes. Additionally, training took place during the off-season, and each athlete received individual nutritional counseling, ensuring optimal energy intake. Moreover, the combination of high-volume training sessions (e.g., 3933.3 kg for the criterion exercises per training session) and high protein intake (e.g., ~1.7 g/kg of body mass) could potentiate lean mass accrual. Furthermore, while the duration was different, our results are in agreement with Kraemer et al. [19], who reported significant increases in lean body mass (3.4 kg, ~5.8%) in division 1 female tennis players following 4 months of periodized ST. Moreover, it should be addressed that different body composition assessments (i.e., DEXA vs. Skin fold calipers) were used, and comparisons between these two investigations should be taken with a degree of caution. The lack of differences between groups with regard to body composition was likely due to similar training volumes and energy intake between groups.

With regard to lower body power measurements, the baseline CMJ height reported in our investigation is similar to previous research conducted on division II female volleyball players (e.g., 33.4 ± 4.3 cm, 31.8 ± 4.6 cm) [20]. Surprisingly, despite significant strength and power improvements, neither group demonstrated improved jump heights. While, the athletes in our investigation may be classified as moderately strength-trained (1RM: total body mass ratio = 1.15) when considering that jumping is critical to key sport skills (i.e., serving, spiking, and blocking), specific jump training may be necessary to see additional improvements [21]. Additionally, it is important to mention that both jump tests (CMJ & SJ) were performed with hands on hips to isolate lower body force production and mitigate potential variations in form [22]. However, as jumping with hands on hips is not common in volleyball, this lack of specificity could have limited our ability to detect improvements. For agility, the average time at baseline was similar to what has previously been reported in division I female volleyball players (e.g., 11.2 ± 0.4 s, 11.1 ± 0.2 s respectively) [23]. Furthermore, both PVBT and OTL significantly decreased time (i.e., improved performance) by 1.8% and 2.1%, respectively suggesting that both groups responded similarly to training. The lack of difference between groups can be attributed to the similar strength and power adaptations.

Previous literature has reported strength gains of 14.1 kg and 4.1 kg on the BS and BP exercises, respectively, following an off-season strength-training (ST) program in division I college female volleyball athletes [24]. Our results are in agreement with the aforementioned study, as we demonstrated average increases of 16.6 kg and 4.1 kg in 1RM on the BS and BP exercises respectively. Concerning the DL exercise, Arazi et al., [25] reported an average increase of 2.0 kg following an 8-week ST regimen in female volleyball players, whereas we demonstrated an average increase of 16.0 kg. The inconsistencies of strength gains between studies could be a result of different training backgrounds prior to training, as well as the training stimulus itself. It is important to address the differences between the VBT regimen reported in our study and traditional percentage-based training regimens reported in a majority of the previous literature. First, the load for both groups was auto-regulated on a set to set basis; therefore, if an athlete was not capable of moving the estimated load in the required velocity range (i.e., 0.85 m·s^−1^) the load would be adjusted to meet their level of performance on that given day. This concept of “auto-regulation” has previously been shown to improve training adaptations, and may further explain the magnitude of strength gains reported in our study [26,27,28]. Moreover, Randell et al. [7] reported that the addition of instantaneous feedback (i.e., peak velocity) to a VBT protocol improved training adaptations compared to a non-feedback condition. Thus, it is likely that a combination of auto-regulatory techniques and instantaneous feedback contributed to a greater magnitude of response to the training regimen across both groups in our investigation. The similar strength adaptations between groups suggest that both PVBT and OTL are effective for inducing gains in maximal strength in female college volleyball players. Regarding the significant difference demonstrated in the deadlift exercise, we do not have a plausible explanation of why this occurred. As there is a lack of data on different VBT regiments and their effects on neuromuscular adaptations, future studies should be conducted to gain further insight.

While maximum strength is considered a critical attribute for success in sports [29], the importance of a preliminary strength block for strength development has recently been under question [14]. In fact, Loturco et al. [30] demonstrated similar strength and power adaptations in elite Brazilian soldiers (i.e., army special forces) undergoing nine weeks of either a traditional strength training progression with a preliminary strength block, or maximum power training (OTL). The progression for traditional strength training was as follows; 1st-mesocycle: 50–80% RM on the BS exercise, 2nd-mesocycle: 30–60%RM on the jump squat exercise, 3rd-mesocycle consisted of CMJ training. In contrast, OTL trained at 45–65% of their 1RM for the BS and jump squat respectively. After the training period, both traditional ST and OTL improved maximum strength on the BS (24.6% and 26.2%, respectively) and 20-m sprint performance (14.5% and 11.6%, respectively). More recently, Loturco et al. [14] compared the effects of traditional strength training versus OTL over the course of a 6-week, in-season training block in elite soccer players. Traditional ST trained at 60–90%RM (~0.67, m·s^−1^ 0.58 m·s^−1^, 0.46 m·s^−1^, 0.40 m·s^−^1) for 4 weeks on the half-squat exercise followed by 30% 1RM (1.2 m·s^−1^) on the jump squat exercise for 2 weeks. In contrast, OTL trained at ~ 1.0 m·s^−1^ on the jump squat exercise for 6 weeks. The results demonstrated similar improvements in BS 1RM (Traditional 8.1% vs. OTL 7.5%) and greater increases in sprinting speed in the OTL compared to traditional ST (traditional 2.3% vs. OTL 5.9%). Our results are in agreement with the aforementioned studies, suggesting that training with loads capable of maximizing power output (i.e., optimum power load) is an efficient method for enhancing strength and power capacities in athletic populations. Lastly, both groups demonstrated a similar increase in measures of internal load (RPE) throughout training. Thus, our data suggest that the internal response to PVBT and OTL is similar over a short training period (i.e., 7-weeks).

The lack of differences in strength and power adaptations between PVBT and OTL in our investigation may be explained by several factors. First off, both groups trained with similar volume loads, exercise selection, and frequency. In fact, the only difference between conditions was the intensity (i.e., velocity) prescribed during the first four weeks of training (e.g., PVBT: 0.55–0.70 m·s^−1^ vs. OTL 0.85–0.90 m·s^−1^). Nevertheless, it is interesting to note the similar adaptations between groups despite the lack of a strength block for the OTL group. Collectively, these findings question the importance of a preliminary strength block. If given a short time period (6–8 weeks) to develop multiple physical attributes, it appears that OTL is equally as effective in various athletic populations (i.e., military, soccer, volleyball athletes).

This study has inherent limitations. First, our low sample size (*n* = 15 total) and relatively short training period (7 weeks) make it difficult to extrapolate these findings to longer training periods. Second, there was no true control group (i.e., court practices only). Nevertheless, it is worth noting that the absence of a control group is commonplace in studies performed with competitive athletes, such as the college volleyball players investigated here. It should also be mentioned that while accessory exercises are common in collegiate strength and conditioning regimens, these could have potentially weakened differences between groups.

## 5. Conclusions

In conclusion, our results demonstrated that both VBT progressions were effective for eliciting positive changes in body composition and performance parameters. It is evident that developing sufficient levels of power output is advantageous for sport performance. While the optimal methods for implementing OTL have not yet been identified, it appears that it is an effective method for developing strength and power. Strength and conditioning professionals should first consider the demands of their sport and select the appropriate exercises accordingly. Next, if considering implementing OTL, they should consider the optimal velocity zone for each exercise, as these many vary. Lastly, the optimal moment to program OTL in an athlete’s overall development has yet to be eluded to. However, our data suggest that it is effective for moderately strength-trained athletes in the off-season period. Future research should aim to investigate the effects of baseline strength levels and response to OTL, as well as its implementation in different periods of the competitive cycle.

## Figures and Tables

**Table 1:** Velocity progressions (m·s^−1^).

	PVBT			OTL		
Week	Day 1—Back Squat	Day 2—Bench Press	Day 3—Deadlift	Day 1—Back Squat	Day 2—Bench Press	Day 3—Deadlift
Week 1	0.55	0.55	0.55	0.85	0.85	0.9
Week 2	0.6	0.6	0.6	0.85	0.85	0.9
Week 3	0.65	0.65	0.65	0.85	0.85	0.9
Week 4	0.7	0.7	0.7	0.85	0.85	0.9
Week 5	0.85	0.85	0.85	0.85	0.85	0.9
Week 6	0.9	0.9	0.9	0.85	0.85	0.9
Week 7	1.0	1.0	1.0	0.85	0.85	0.9

PVBT—Progressive velocity-based training, OTL—Optimum training load.

**Table 2:** Set and repetitions progressions.

	PVBT			OTL		
Week	Day 1—Back Squat	Day 2—Bench Press	Day 3—Deadlift	Day 1—Back Squat	Day 2—Bench Press	Day 3—Deadlift
Week 1	4 × 3 6 × 2	4 × 3 6 × 2	10 × 2	4 × 4 6 × 3	7 × 5 3 × 4	4 × 4 6 × 3
Week 2	7 × 3 3 × 2	7 × 3 3 × 2	4 × 3 6 × 2	4 × 4 6 × 3	10 × 5	10 × 3
Week 3	11 × 3	12 × 3	6 × 3 6 × 2	10 × 4	8 × 6 4 × 5	12 × 3
Week 4	12 × 3	5 × 4 8 × 3	8 × 3 4 × 2	12 × 4	9 × 6 4 × 5	12 × 3
Week 5	4 × 4 8 × 3	6 × 6 4 × 5	8 × 3 4 × 2	12 × 2	10 × 6	6 × 2 6 × 1
Week 6	4 × 4 8 × 3	12 × 6	13 × 2	13 × 4	12 × 6	13 × 1
Week 7	2 × 5 10 × 4	10 × 5	9 × 3 1 × 2	12 × 2	10 × 5	10 × 3

PVBT—Progressive velocity-based training, OTL—Optimum training load.

**Table 3:** Accessory exercises.

Accessory Exercises		
Day 1	Day 2	Day 3
Barbell Squat Jumps	Supine Medicine Ball Press	Trap Bar Squat Jumps
Hurdle Jumps	TRX Inverted Row	Seated Dumbbell Box Jumps
Barbell Lateral Lunge	Pull Ups	Barbell Lateral Step-Up & Over
Rear Foot Elevated Split Squats	Side Plan Cable Row	Landmine Single Leg Romanian Deadlift
